# The Impact of Different Antibiotic Regimens on the Emergence of Antimicrobial-Resistant Bacteria

**DOI:** 10.1371/journal.pone.0004036

**Published:** 2008-12-29

**Authors:** Erika M. C. D'Agata, Myrielle Dupont-Rouzeyrol, Pierre Magal, Damien Olivier, Shigui Ruan

**Affiliations:** 1 Division of Infectious Diseases, Beth Israel Deaconess Medical Center, Harvard Medical School, Boston, Massachusetts, United States of America; 2 Laboratoire d'Ecotoxicologie des Milieux Aquatiques, Université du Havre, Le Havre, France; 3 Laboratoire de Mathématiques Appliquées, Université du Havre, Le Havre, France; 4 Laboratoire d'Informatique, de Traitement de l'Information et des Systèmes, Université du Havre, Le Havre, France; 5 Department of Mathematics, University of Miami, Coral Gables, Florida, United States of America; Charité-Universitätsmedizin Berlin, Germany

## Abstract

**Backgroud:**

The emergence and ongoing spread of antimicrobial-resistant bacteria is a major public health threat. Infections caused by antimicrobial-resistant bacteria are associated with substantially higher rates of morbidity and mortality compared to infections caused by antimicrobial-susceptible bacteria. The emergence and spread of these bacteria is complex and requires incorporating numerous interrelated factors which clinical studies cannot adequately address.

**Methods/Principal Findings:**

A model is created which incorporates several key factors contributing to the emergence and spread of resistant bacteria including the effects of the immune system, acquisition of resistance genes and antimicrobial exposure. The model identifies key strategies which would limit the emergence of antimicrobial-resistant bacterial strains. Specifically, the simulations show that early initiation of antimicrobial therapy and combination therapy with two antibiotics prevents the emergence of resistant bacteria, whereas shorter courses of therapy and sequential administration of antibiotics promote the emergence of resistant strains.

**Conclusions/Significance:**

The principal findings suggest that (i) shorter lengths of antibiotic therapy and early interruption of antibiotic therapy provide an advantage for the resistant strains, (ii) combination therapy with two antibiotics prevents the emergence of resistance strains in contrast to sequential antibiotic therapy, and (iii) early initiation of antibiotics is among the most important factors preventing the emergence of resistant strains. These findings provide new insights into strategies aimed at optimizing the administration of antimicrobials for the treatment of infections and the prevention of the emergence of antimicrobial resistance.

## Introduction

Antimicrobial resistance among bacteria has become a worldwide public health threat [1]–[3]. Despite substantial interventions aimed at preventing the emergence and spread of antimicrobial-resistant bacteria, the rates continue to rise rapidly [4]. The geographic locations affected by antimicrobial resistance are mounting [2]. The economic impact of antimicrobial resistance is substantial [1]. Infections caused by these antimicrobial-resistant bacteria are associated with substantially higher mortality rates, longer hospital stays and greater hospital costs, compared to infections caused by antimicrobial-susceptible bacteria [5], [6]. In 1998, it was estimated that the annual cost of antimicrobial resistance in hospitals due to *Staphylococcus aureus* was already $122 million and of nosocomial infections was $4.5 billion [1], [7]. A recent estimate showed that there were 18,650 deaths in patients with invasive methicillin-resistant *S. aureus* in the United Sates in 2005, exceeding the total number of deaths due to HIV/AIDS in the same year [8].

In the last decade, mathematical models have been increasingly used as tools to identify factors responsible for observed patterns of antimicrobial resistance, to predict the effect of various factors on the prevalence of antimicrobial resistance, and to help design effective control and intervention programs [9]–[16]. We also refer to the surveys on this topic [14], [15], [17]–[19]. Most of these studies have used differential equations models, which aggregate patient and health-care worker populations into compartments such as colonized or uncolonized patients and contaminated or uncontaminated health-care workers. Interventions proposed by these studies have focused on reducing the transmission of antimicrobial-resistant bacteria between patients thereby preventing *de novo* acquisition. Antimicrobial treatments have also been evaluated in some of these studies. For example, Bonhoeffer et al. [10] considered two models for treatment and resistance with a single and two drugs, respectively. They found that when more than one antibiotic is employed, sequential (cycling) use of different antibiotics is not as good as that with a combination of antibiotics. Bergstrom et al. [11] further developed a mathematical model to study the efficacy of cycling program and found that cycling is unlikely to reduce the spread of antimicrobial resistance. Se also [49], [50].

One of the main limitations in providing guidance with antibiotic administration is the paucity of data regarding optimal duration of therapy and patterns of use which would reduce the emergence and spread of resistant bacteria. This lack of data is due to the fact that patient-oriented clinical studies cannot fully address the multitude of factors that are involved in the treatment of infections, the emergence of resistance and the role of the immune system in eradicating the infection. D'Agata et al. [12] integrated an individual-based model and a deterministic model to provide a quantitative analysis of the emergence and spread of antimicrobial resistant bacteria and demonstrated that early initiation of treatment and minimization of its duration mitigates antimicrobial resistance epidemics in hospitals.

Mathematical models have also developed to study pharmacodynamics of various antimicrobial therapies [20]–[23]. Lipsitch and Levin [22] presented a simple mathematical model of pharmacokinetics and bacterial population dynamics that is designed to address the problem of suppressing the emergence of resistance during treatment. They restricted their consideration to treatment with bactericidal antibiotics and the evaluation of resistance by mutation. Via mathematical modelling and computer simulations, the effect of mutation in antimicrobial resistance has further investigated by Levin and Rozen [23] who showed non-inherited resistance could extend the duration of antimicrobial treatment, cause treatment failure and promote the generation and ascent of inherited resistance in treated patients.

Bacteria can also develop antimicrobial resistance through the acquisition of new genetic material from other resistant organisms, such as horizontal gene transfer [24], [25]. The widespread dissemination of antimicrobial resistance genes are the results of improper and excessive administration of antibiotics, combined with the ready bacterial ability to transfer antimicrobial resistance genes through plasmids and transposons and the presence of large transfer communities such as hospitals [24]. Compared to the mathematical modelling on population dynamics of the antimicrobial resistance bacteria, there are few models describing the horizontal transfer of antimicrobial resistance genes [24], which could help better understand the mechanisms of antimicrobial resistance in bacteria and provide more effective treatment.

Multidrug-resistant bacteria can colonize specific sites in the host and evade immune surveillance [26]. The nature of the host immune response to multidrug-resistant bacterial infection is complex. To the best of our knowledge, the combined effect of immune response, horizontal gene transfer and antibiotic treatment has not been modelled and explored. In this article, we develop a new mathematical model which incorporates three key aspects in the emergence of resistance caused by antibiotic exposure: the response of the host's immune system, horizontal transfer of resistance genes and patterns of antibiotic treatment regimens. Specifically, we want to propose a model for the bacterial population and study the within-host dynamics that would provide critical information regarding the optimal regimens for antibiotics administration in the treatment of infections in order to prevent the emergence of antimicrobial resistant bacteria. By mathematical modelling and numerical simulations, we demonstrate the importance and significance of the necessary length of antimicrobial treatment, the early initiation of treatment, and the combination of antibiotics in preventing antimicrobial resistant bacterial infections in treated patients.

## Methods

### (A) Model with immune response and single antibiotic treatment

We first present an ideal case of a homogenous bacterial population within a host treated by an antimicrobial agent. Since a growing bacterial population will eventually saturate due to the limitation of nutrients and space, we use the logistic growth to describe the population dynamics of the bacteria. Also assume that the bacteria are killed by the antimicrobial agent at a constant rate proportional to the density of the bacteria population.

Let *B(t)* be the number of bacteria at time *t*. Let δ (day^−1^) be the division rate and μ (day^−1^) the mortality rate due to antibiotic treatment. So *λ* = δ−μ is net growth rate and *λκ* is the carrying capacity of the bacteria, the term 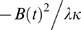 describes the limitation of space and food available to the bacteria. The bacterium *Escherichia coli* is chosen for this model since baseline parameters pertaining to its biology are available from the literature. For *E. coli*, the average *in vivo* doubling time (AV) is 0.4 day, based on data from several studies [27], [28], [29]. The growth rate is *λ* = ln2/ AV = 2.7726 day^−1^. The maximum number is set at 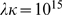 bacteria [12]. Thus, 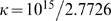.

Now we consider the response of the host's immune system to the invading bacteria. The innate immunity is characterised by a rapid action of the host's effectors cells including leukocytes, which will limit the multiplication of bacteria and destroy them. Assume that the killing rate of bacteria by leukocytes satisfies the Monod function [21], [30]. In our model, the minimal infecting dose, or threshold of bacteria required to overcome the immune system, will be addressed. This value implies that above the threshold, the immune response is ineffective against bacterial growth and the infection progresses.

For the immune system to be ineffective and the infection to progress, the bacterial population need to be above the minimal infecting dose, or threshold, which is related to the number of phagocytes. Indeed, we can consider the following equation:
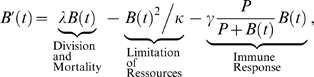
(1)where *P* is the equilibrium number of activated phagocytes, and *γ* (day^−1^) is the maximal killing rate of bacteria by activated phagocytes. We assume that the number of phagocytes remains constant. The main consequence of this assumption is that the threshold of invasion and the threshold of bacterial eradication are the same [21]. For other values see Table 1.

**Table 1:** List of Parameters and their Values.

Symbol	Interpretation	Value	Units	References
*λ*	Growth rate of bacteria	2.7726 (without treatment)(i)	day^−1^	[27], [28], [29]
		2.7726 −1.9 with treatment A (sensitive to A)		
		2.7726 −2.1 with treatment B (sensitive to B)		
δ	Division rate of bacteria	2.7726	day^−1^	This study(ii)
*μ*	Mortality rate of sensitive bacteria	0 without treatment	day^−1^	This study
		1.9 with treatment A (for sensitive to A)	day^−1^	
		2.1 with treatment B (for sensitive to B)		
*κ*	*λκ* is the carrying capacity of bacteria	10^15^/2.7726(iii)		This study
γ	Killing rate of Phagocytes	33.6038(iii)	day^−1^	This study
P	Total Number of Phagocytes	332711(iii)		This study
τ	Recombination rate	10^−3^	day^−1^	[41], [43]
p	Probability that a plasmid is lost during the division without antibiotic pressure	0.4(iv)		This study

(i) In the work from [31], the *E. coli* generation time *in vivo* in the gastrointestinal tract was approximately 60 min (*λ* = 16.6622 day^−1^) and was quasi-static in the lumen content. However, the authors state that these results are specific to their experimental conditions, without competition with other bacteria. In our model, we chose a longer generation time based on (a) other studies [27]–[29]; and (b) the relevance of our simulations. Indeed, if *λ* is set at 16.6622 day^−1^, the bacterial growth was at a rate that antibiotic therapy was totally inefficient, even for susceptible bacteria.

(ii) In this study we assume that the mortality rate of bacteria in absence of antibiotics is negligible.

(iii) These parameters have been adjusted to maintain an infection threshold of 10^6^ bacteria [32] and a rate of invasion comparable to previous studies [12].

(iv) The probability that a plasmid is lost during the division without antibiotic pressure depends on the bacteria and plasmid size. According to several studies, we chose an average parameter representative of *E. coli* and the common size of plasmid bearing resistance genes [33]–[36]. This probability may vary from 0 to 1 among 50 generations cell. In this model, this parameter was set at a value to provide adequate simulations.

Model (1) has at most three non-negative equilibria. 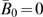 is always an equilibrium, which is stable if *λ* = δ−μ>0 and unstable if *λ* = δ−μ<0. There eventually exist two other equilibria which are solutions of the quadratic equation 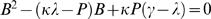. It has two roots 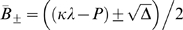 if 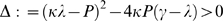, where 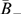 corresponds to the invasion threshold and 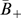 corresponds to the maximal bacterial load. In practice, we fix 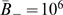 [32] and 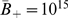 corresponding to the experimental value obtained in previous studies [12]. Notice that with parameter values in Table 1, 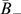 is unstable and 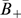 is stable. Thus, the asymptotic behavior of solutions of Model (A) depends on the location of the initial value. If the initial bacterial load is between 10^6^ and 10^15^, then the solution will increase and approach 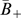 as time involves. However, treatment can change the dynamics (see simulations in Results).

### (B) Model with resistant strains to one antibiotic through horizontal gene transfer

Antimicrobial resistance among bacteria can be intrinsic or acquired either through *de novo* mutations or *via* the acquisition of antimicrobial-resistance genes [25]. The latter occurs through horizontal gene transfer of mobile genetic elements including plasmids, integrons and transposons^37^. Acquisition of resistance genes is among the main mechanism of antimicrobial resistance among *E. coli* isolates [38]–[40].

Let 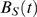 and *B_R_*(*t*) denote the population levels of antibiotic-sensitive (plasmid free) and antibiotic-resistant (plasmid bearing) bacteria at time *t*, respectively, so that 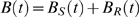 is the total bacterial load in a host. Thus, 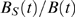 is the fraction of bacteria that are antibiotic-sensitive and 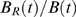 is the fraction of bacteria that are antibiotic-resistant. Let *τ* be the recombination rate (i.e. horizontal gene transfer rate) of plasmid free and plasmid bearing to plasmid bearing. Then 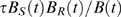 represents the recombination process. Let *p* be the probability that a plasmid is lost, which varies after 50 generations from 0, 40 or 100% [33]–[35] and depends on the type of plasmid, its stability and copy number, and the presence of the antibiotic pressure. In practice this leads to the estimation of 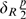 as the reversion rate of plasmid bearing to plasmid free, where 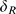 is the division rate of the antibiotic-resistant bacteria. Let 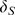 be the division rate of the antibiotic-sensitive bacteria, 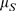 and 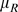 be the mortality rates of plasmid free and plasmid bearing bacteria, respectively.

Extending the model of Webb et al. [16] to include the immune response, we obtain the following:
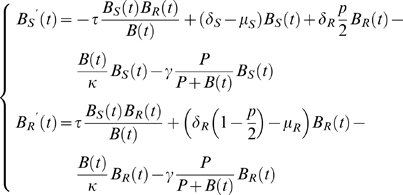
(2)The rate of horizontal gene transfer occurs at a rate ranging from 10^−1^ to 10^−6^ genes per cell per generation [41]–[43]. In this model, we assume that gene transfer occurs at a rate of 10^−3^/day. Since plasmid-bearing bacteria have a longer generation time than plasmid-free bacteria, we assumed a 40% increase in generation time among the plasmid-bearing bacteria [36], [44]. In the absence of antibiotic pressure, plasmids carrying antimicrobial resistance genes are lost to minimize costs associated with replication and conservation, as shown in several studies [33]–[36]. In our model, plasmid loss during bacterial replication is set at 10^−5^ per cell and per generation [36], [45], [46]. In absence of antibiotics, we can assume that 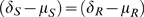. In this case *B(t)* satisfies Model (1). So we observe that 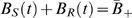.

Note that we have extinction when 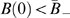 or invasion when 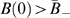 and (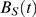, *B_R_*(*t*)) converges to (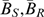) as time *t* increases, where 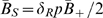 and 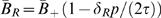. The proportion of antibiotic-susceptible and -resistant bacteria can therefore be quantified in function of the parameter in absence of antibiotics.

### (C) Model with different antibiotic treatments and multidrug-resistant (MDR) strains

Finally we consider the situation when the bacteria are multidrug-resistant. We will model different antibiotic treatments and study the combined treatment regimens. Denote

*B_S_(t)*–the number of antibiotic-sensitive (plasmid-free) bacteria at time *t*
*B_A_(t)*–the number of bacteria resistant to antibiotic *A* but not resistant to antibiotic *B* (i.e. bacteria bearing only plasmid *A*)
*B_B_(t)*–the number of bacteria resistant to antibiotic *B* but not resistant to antibiotic *A* (i.e. bacteria bearing only plasmid *B*)
*B_AB_(t)*–the number of bacteria resistant to both antibiotics *A* and *B* (i.e. bacteria bearing both plasmids *A* and *B*) .

Considering exposure to two antibiotics and generalizing the Model (2) which includes the effect of the saturation due to food or space limitation, and the effect of immune system, we have the following model:
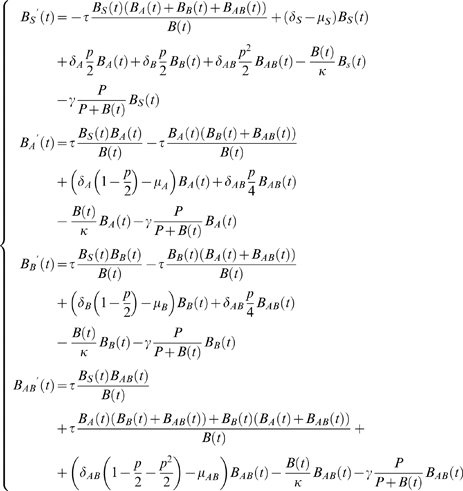
(3)To simplify, we will assume that 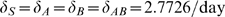 and the mortality rates are shown in Table 1.

## Results

By carrying out numerical simulations of Models (1)–(3), we study the impact of several factors, such as the immune response, duration of antimicrobial therapy, initiation and interruption of treatment, delay and sequential antimicrobial regimens, and combination of antimicrobial therapies, on the progress of an infection within a host. Note that in all simulations, the initial values are chosen near the equilibrium values of these models.

### (A) Immune system response and duration of antimicrobial therapy

**Figure 1:**
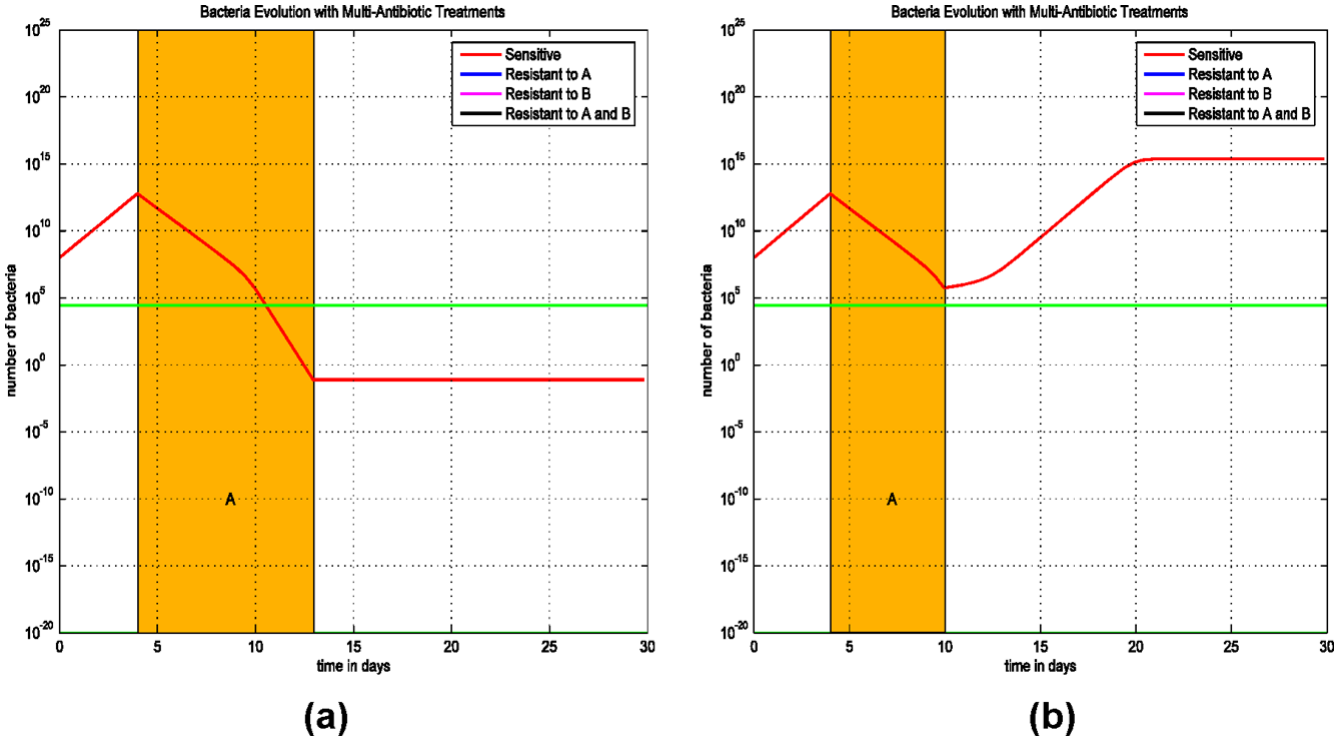
Simulations of Model (1) on the duration of antimicrobial treatment and its effects on infection progression. The initial value is chosen between 10^6^ and 10^15^, so the solution increases initially. The green line represents the threshold of bacterial load above which an infection develops. Below this threshold, the phagocyte density is sufficient to prevent the progression to infection. (a) Treatment starts at the 4^th^ day after an infection and lasts for 9 days. The bacterial load decreases to below the threshold and the infection is prevented. (b) Treatment starts at the 4^th^ day after an infection and lasts for only 6 days. The bacterial load decreases to close to the threshold, but stays above it and increases again. The infection becomes more progressive.

The first simulation (Model (1)) demonstrates the importance of longer antimicrobial therapies required to prevent the progression of an infection. When the bacterial load is below the minimal infecting dose, the immune system is effective in killing bacteria and the patient does not develop an infection. When the duration of an antimicrobial therapy is for 9 days, the bacterial load is reduced below the threshold and progression of the infection is prevented. However, a shorter antimicrobial therapy of 6-day courses does not reduce the bacterial load to below the threshold and therefore an infection is not prevented (Figure 1).

### (B) Initiation and interruption of treatment and resistant strains to one antibiotic

The second simulation (Model (2)) addresses the issue of the importance of the timing of antibiotic initiation in the progression to an infection. The model includes the susceptible strain and a strain that is resistant to antibiotic A. Antibiotics are administered for a total of 9 days to optimize the duration of therapy, as shown in Figure 1.

**Figure 2:**
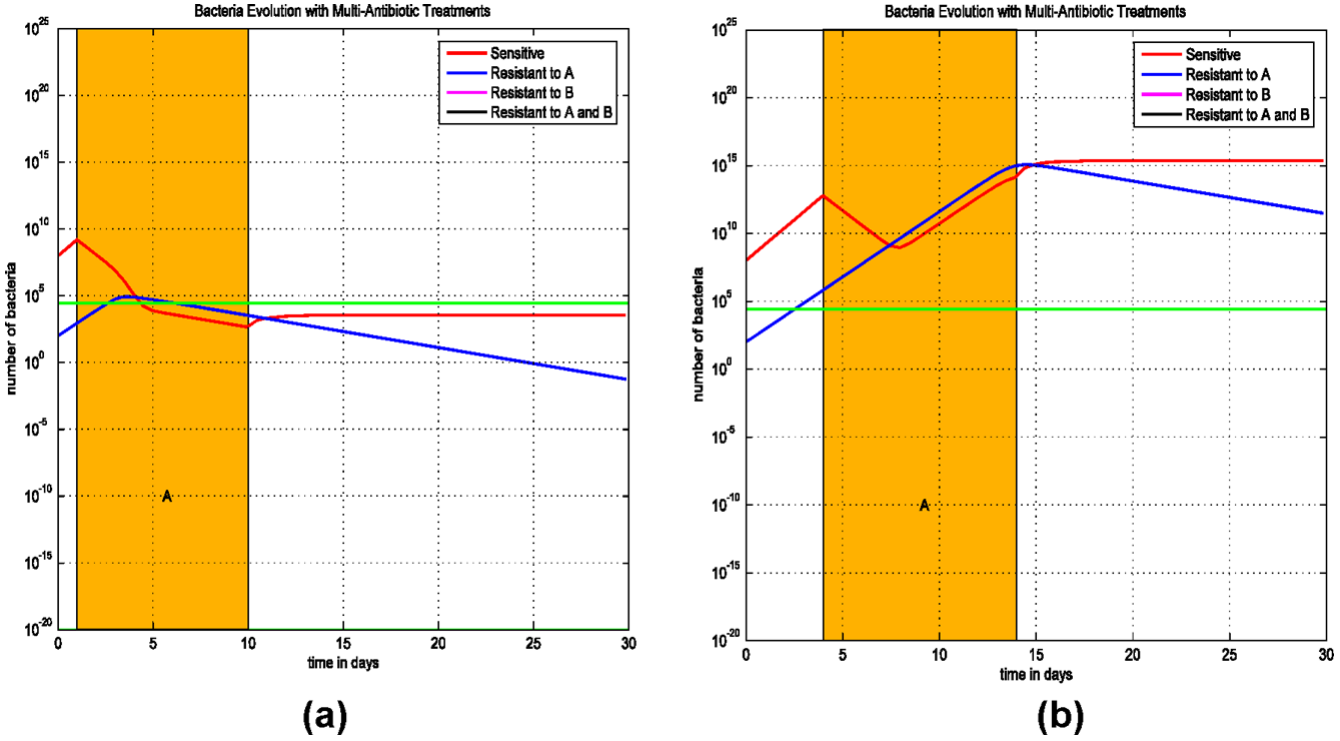
Simulations of Model (1) on the initiation of an antimicrobial therapy and infection progression. (a) Treatment starts at the 1^st^ day after an infection and lasts for 9 days. The bacterial load decreases to below the threshold and the infection is prevented. (b) Treatment starts at the 3^rd^ day after an infection and lasts for 9 days. The bacterial load decreases slightly, but stays above the threshold and increases even during the treatment.

In Figure 2(a), the treatment starts at the first day of an infection and in Figure 2(b), treatment is delayed and starts at the third day of the infection. The treatment occurs with antibiotic A which is ineffective against the resistant strain A. The simulations show that if the initiation of a therapy is delayed the infection will progress. If the antimicrobial therapy starts early, the infection can be treated successfully, with both the susceptible and resistant strains.

**Figure 3:**
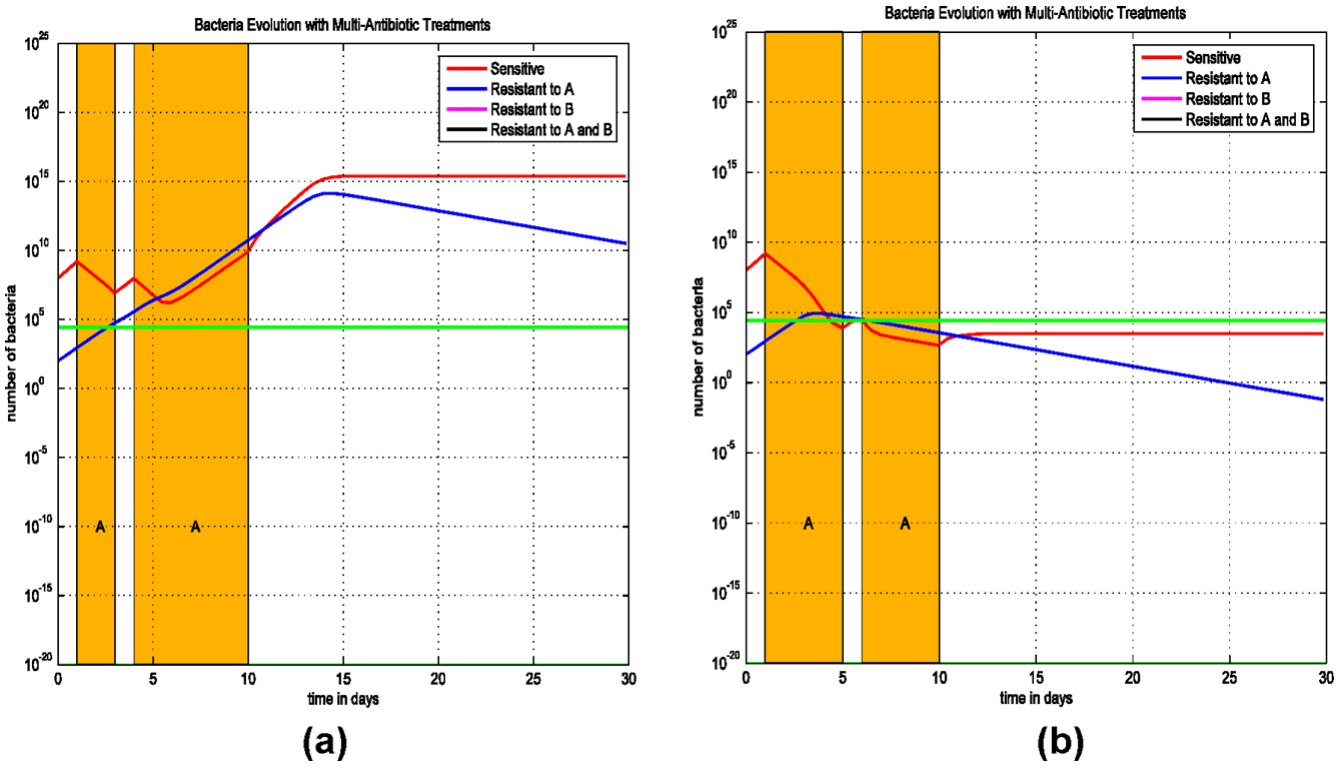
Simulations of Model (2) on the interruption of treatment and infection progression. (a) Treatment is interrupted for 1 day at day 3 and continues for the rest of the 9-day therapy. The bacterial load does not change much and increases afterward, the infection is progressive. (b) Treatment is interrupted for 1 day at day 5 and continues for the rest of the 9-day therapy. The bacterial load decreases steadily and the infection can be prevented.

The third simulation (Model (2)) addresses the issue of treatment interruption and its impact on the progression of an infection. The importance of initiating appropriate antibiotic therapy at the start of infection is addressed in these simulations. In both Figures 3(a) and 3(b) the treatment duration is for 9 days, but in Figure 3(a) the treatment is interrupted for one day at day 3 and in Figure 3(b) the interruption occurs at day 5. These simulations demonstrate that early interruption of therapy results in the progression of an infection with the resistant strain since the bacterial load of the resistant strain exceeds the threshold of the minimum infecting dose prior to treatment interruption.

**Figure 4:**
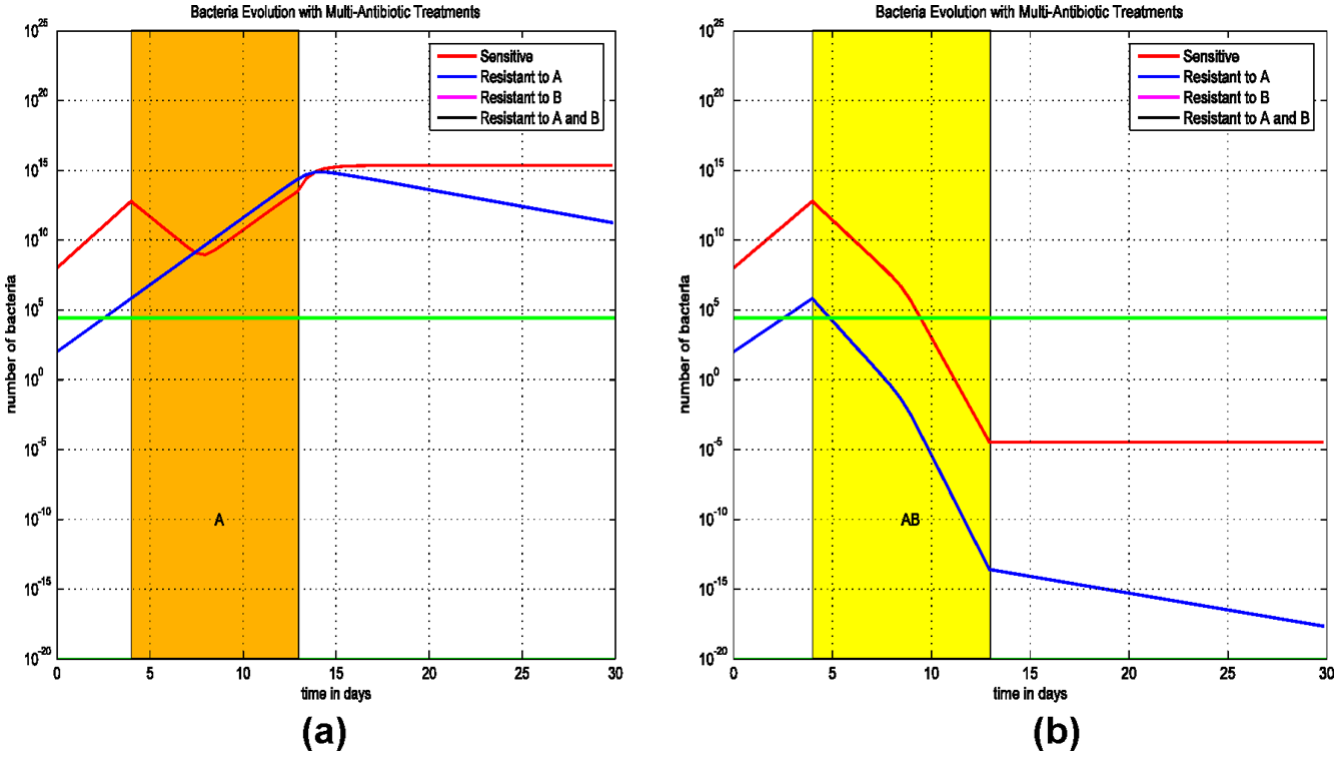
Simulations of Model (3) on multiple antibiotic therapies. In both simulations, treatment starts at the 4^th^ day after the infection and lasts for 9 days. (a) If antibiotic A fails in the first therapy, then a second treatment with antibiotic B has to be administrated to prevent the infection. (b) A combination of both antibiotics A and B can bring the bacterial level below the threshold and the infection can be prevented.

We assume that the patient is harbouring a sensitive strain (AsBs) and a strain that is resistant to antibiotic A but susceptible to antibiotic B (ArBs). The effects of varying treatment sequences with antibiotics A and B are simulated in Figure 4. Treatment with antibiotic A is ineffective and the infection progresses, but subsequent treatment with antibiotic B is effective in eradicating the infection (Figure 4(a)). Concurrent therapy with antibiotics A and B is more effective as the patient is treated with an effective antibiotic against ArBs (Figure 4(b)).

### (C) Delay and sequential antibiotic regimens on the emergence of MDR strains

The impact of early initiation of therapy is addressed in the final model (Model (3)) for treating two-drug resistant strains, since this treatment was effective in eliminating the single-drug resistant strain (Figure 2(a)). We assume that the patient is colonized with three *E. coli* populations: a strain that is sensitive to both antibiotics A and B (AsBs), a second strain that is sensitive to antibiotic A and resistant to antibiotic B (AsBr), and a third strain which is resistant to antibiotic B and sensitive to antibiotic A (AsBr). The impact of concurrent or sequential therapies with antibiotics A and B and its effect on the emergence of the two-drug resistant strains (ArBr) is assessed.

**Figure 5:**
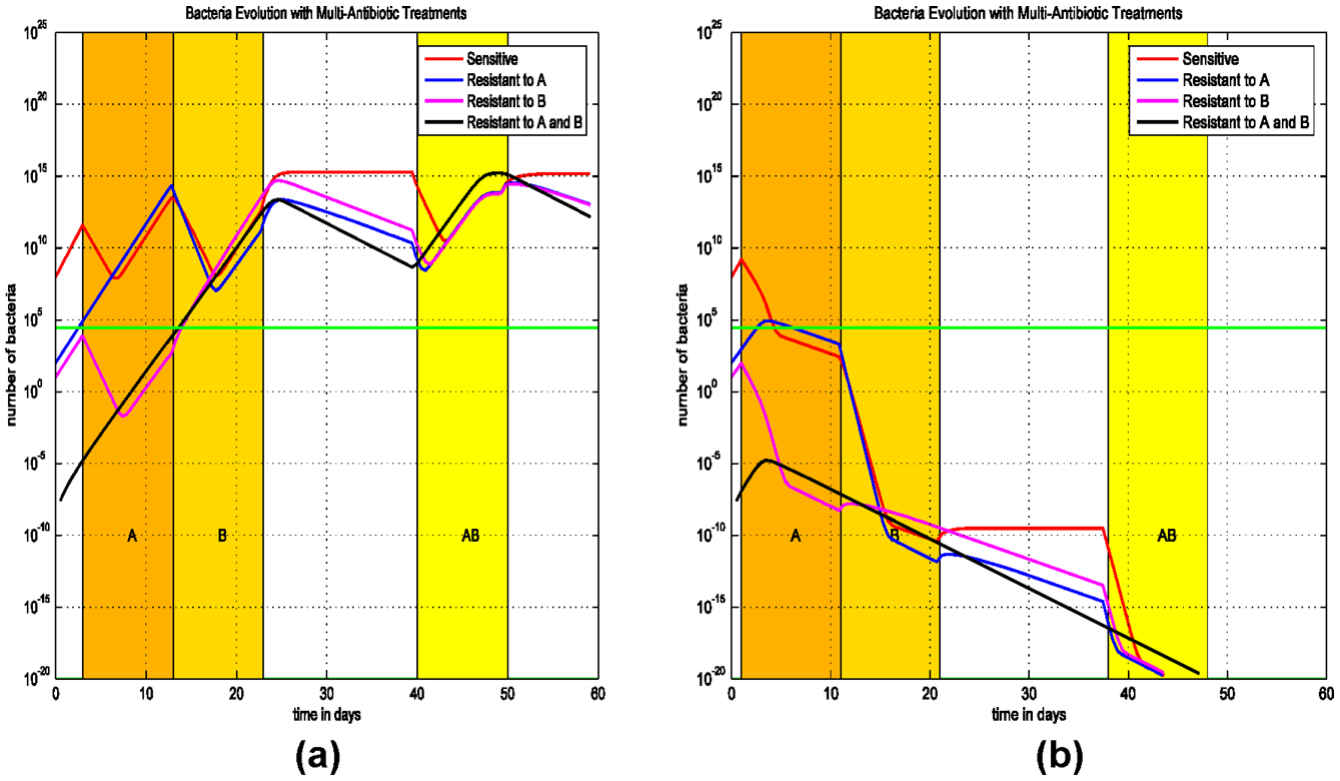
Simulations of Model (3) on sequential treatment with antibiotics A and B. (a) Treatment with antibiotic A starts at the 3^rd^ day after the infection and lasts for 10 days, right after that antibiotic B is administrated for 10 days, and at the 40^th^ day both antibiotics A and B are used for 10 days. (b) Same therapies except that the first treatment starts at the 1^st^ day after the infection.

We consider two scenarios: (i) The patient is treated with the antibiotic A and than immediately with the antibiotic B, followed by both antibiotics simultaneously (Figure 5(a)). (ii) Treatment starts at the first day after the infection (Figure 5(b)). Simulations demonstrate that early initiation of therapy, on day 1, can eliminate the two-drug resistant strain ArBr irrespective of the sequence of antibiotic therapies (Figure 5(b)). In this scenario, the bacterial load of ArBr always remains below threshold. Thus, early initiation of therapy and combined antimicrobial therapy are the key measures to prevent and control infections with multi-drug resistant strains.

**Figure 6:**
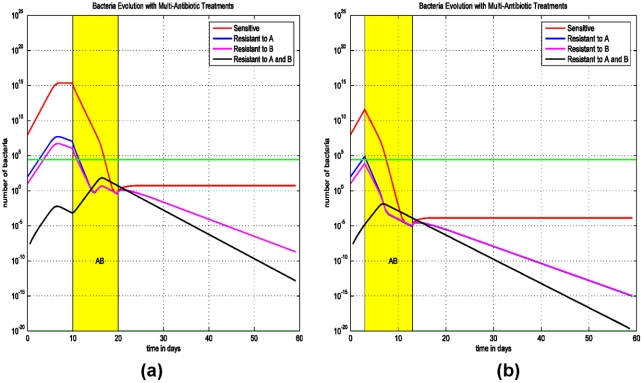
Simulations of Model (3) on early initiation of therapy and evolution of the bacteria population with sensitive and bacteria resistant to antibiotics A and (or) B. (a) A combined therapy with both antibiotics A and B did not start until the 10^th^ day after the infection. After 10 days, the treatment prevents the progression of the infection. (b) A combined therapy of both antibiotics A and B starts at the 3^rd^ day after the infection and lasts for 10 days.

To further simulate the early initiation of therapy and the effect of combined therapy, in Figure 6(a) the patient is treated on the 3^rd^ day after infection with both antibiotics A and B simultaneously. In Figure 6(b) the patient is treated on the 10^th^ day after infection with both antibiotics A and B simultaneously.

In these simulations (Figures 5 and 6), the antibiotic regimens that eradicate the infection and eliminate the resistant strains are therapies using antibiotics A and B simultaneously. In Figure 5(b) and Figure 6, as opposed to Figure 5(a), the bacterial loads of AsBr and ArBs decrease during therapy with antibiotic A and B, respectively. The decrease in bacterial quantity diminishes the rate of horizontal gene transfer, thereby preventing the emergence of ArBr, despite ineffective therapy against two-drug resistant strain. The efficacy of initiating therapy with both antibiotics simultaneously is persistent even if therapy is delayed (Figure 6(a)). The optimal regimen in treating the two-drug resistant strains is demonstrated in Figure 6(b): early treatment with a combination of two antibiotics.

## Discussion

Antimicrobial exposure is central to the emergence and spread of antimicrobial-resistant bacteria. We have presented a model delineating the interrelated factors of antimicrobial therapy, the immune system and resistance gene transfer, and their effect on the emergence of antimicrobial-resistant bacterial strains. The model delineates novel findings with regards to the timing and sequence of antibiotic therapy in preventing the emergence of resistant strains. First, the model shows that shorter lengths of antibiotic therapy and early interruption of antibiotic therapy provide an advantage for the resistant strains and result in infections caused by these resistant bacteria to progress. Second, the model outlines the optimal antibiotic regimens which prevent the progression of infection with a resistant strain. Our model demonstrates that combination therapy with two antibiotics prevents treatment failure and the emergence of resistant strains, as opposed to sequential therapy. Third, the model shows that a delay in the start of therapy is one of the key factors in promoting the rapid rise in resistant strains. The early timing is even more important than the type of antibiotic regimen since early initiation of antimicrobials will prevent emergence of resistance regardless of whether antibiotics are administered sequentially or concurrently. Using a model system which incorporated population-level factors, D'Agata *et al.* [12] also demonstrated that early initiation of therapy is key to preventing the spread of antibiotic-resistant bacteria within a hospital setting.

Population-level models quantifying the factors promoting the spread of resistant bacteria have been instrumental in identifying the preventive strategies most effective at decreasing cross-transmission between patients [9]–[19]. These models have shown the impact of overall antimicrobial exposure on the spread of resistant bacteria. In our individual-level model, we extend the analysis of antimicrobial exposure and treatment regimen to further our understanding of the type and sequence of antimicrobial exposure to provide guidance on the optimal prescribing patterns.

Clinical studies do not provide conclusive evidence of the benefit of combination therapy in the treatment of infections with the exception of tuberculosis and HIV [47]. In these cases, combination therapy is effective due to the high bacterial burden and high rate of emergence of resistance during therapy through mutations [47]. For other bacteria is remains unclear from clinical studies whether combination therapy provides a benefit in reducing resistance. Thus, current guidelines for improving antibiotic use from the Infectious Disease Society of America and the Society for Healthcare Epidemiology do not recommend use of combination therapy [47]. However, we would like to point out that in clinical practice, when laboratory tests confirm the co-existence of different antimicrobial-resistant bacteria with different antimicrobial susceptibility profiles, combination therapy is used to target each pathogen. In our paper, combination therapy referred to the use of two antimicrobials both of which would have efficacy against the infecting organism. In fact, our model supports the use of combination therapy in preventing the emergence of multiple resistant strains within an individual [10], [11], [19], [22], [48]. The effect of widespread combination therapy on the emergence and spread of resistant bacteria to other patients however was not addressed. Future models should incorporate both individual- and population level analysis to determine the impact of the spread of resistant bacteria within a community or hospital setting.

For the purpose of numerical simulations, in Model (1) we assumed that the growth of bacteria satisfies the specific logistic equation and the immune response satisfies the specific Monod function. It should be pointed out that Model (1) can be generalized to include a general bacteria growth term *g(B)* and a general immune response function *p(B)*, where *g(B)* is continuously differentiable and satisfies *g(0) = g(K) = 0* for a positive constant K, and *g′(0)>0*, *g′(K)<0*. *p(B)* is also continuously differentiable and satisfies *p(B)>0* and *p′(B)>0*. Similar extensions apply to Models (2) and (3).

Our conclusions were mainly based on the numerical simulations of Models (1)–(3). It would be very interesting and helpful to perform qualitative analysis of these models and provide theoretical support for our numerical simulations and thus our conclusions. Studies on the dynamics of these models are under consideration and will be reported somewhere else.
